# Recurrence Plots: a New Tool for Quantification of Cardiac Autonomic Nervous System Recovery after Transplant

**DOI:** 10.21470/1678-9741-2016-0035

**Published:** 2017

**Authors:** Isabela Thomaz Takakura, Rosangela Akemi Hoshi, Márcio Antonio Santos, Flávio Correa Pivatelli, João Honorato Nóbrega, Débora Linhares Guedes, Victor Freire Nogueira, Tuane Queiroz Frota, Gabriel Castro Castelo, Moacir Fernandes de Godoy

**Affiliations:** 1 Faculdade de Medicina de São José do Rio Preto (FAMERP), São José do Rio Preto, SP, Brazil; 2 Universidade de Fortaleza (Unifor), Fortaleza, CE, Brazil

**Keywords:** Heart failure, Transplantation, Autonomic Nervous System

## Abstract

**Objective:**

To evaluate a possible evolutionary post-heart transplant return of autonomic function using quantitative and qualitative information from recurrence plots.

**Methods:**

Using electrocardiography, 102 RR tachograms of 45 patients (64.4% male) who underwent heart transplantation and that were available in the database were analyzed at different follow-up periods. The RR tachograms were collected from patients in the supine position for about 20 minutes. A time series with 1000 RR intervals was analyzed, a recurrence plot was created, and the following quantitative variables were evaluated: percentage of determinism, percentage of recurrence, average diagonal length, Shannon entropy, and sample entropy, as well as the visual qualitative aspect.

**Results:**

Quantitative and qualitative signs of heart rate variability recovery were observed after transplantation.

**Conclusion:**

There is evidence that autonomic innervation of the heart begins to happen gradually after transplantation. Quantitative and qualitative analyses of recurrence can be useful tools for monitoring cardiac transplant patients and detecting the gradual return of heart rate variability.

**Table:** 

Abbreviations, acronyms & symbols	
AF	= Atrial fibrillation
ApEn	= Approximate entropy
CD	= Correlation dimension
DET	= Determinism
DFA	= Detrended fluctuations analysis
ENTR	= Entropy
HRV	= Heart rate variability
LAM	= Laminarity
Lmax	= Maximum line
Lmean	= Average diagonal length
REC	= Recurrence
RPs	= Recurrence plots
RR	= RR intervals
SampEn	= Sample entropy
ShEn	= Shannon entropy
TT	= Trapping time

## INTRODUCTION

Heart failure is a complex clinical syndrome that results in loss of functional or structural blood ejection or ventricular filling^[1]^. Heart transplantation is recognized as the best treatment for refractory heart failure, even in the absence of randomized controlled trials^[2]^.

However, exercise performance after heart transplantation remains low despite the absence of symptoms. The absorption of oxygen during exercise and anaerobic ventilatory threshold are in the predicted range of 50%-70%^[3,4]^.

The cause of exercise intolerance in heart transplant patients is not well known, but there is evidence that it is multifactorial and related to neurohumoral cardiac, vascular, muscle, and lung changes^[4]^. Givertz et al.^[4]^ suggest that the chronotropic incompetence is due to denervation of the heart during surgery and that the innervation occurs about five years after the transplant.

Compared to healthy subjects, heart rate in transplant patients is fixed and linear, and heart rate variability (HRV) is practically absent^[5]^; however, some studies show that, in general, HRV improves over time after transplantation, suggesting the occurrence of re-innervation^[6,7]^.

Cornelissen et al.^[8]^ assessed HRV in 14 supine, resting patients undergoing heart transplantation using an electrocardiogram monitor for 20 minutes, with a first exam around the 13^th^ month after transplantation and again at 141 months. They observed statistically significant increases in HRV in all patients.

Autonomic nervous system homeostasis can be assessed by HRV analysis that, according to Godoy^[9]^, shows the characteristics of a dynamic system and is complex, non-linear, and extremely sensitive to initial conditions. Currently, this is also the scientific concept for the existence of "chaos" hence HRV shows chaotic behavior, as do all systems of the human organism^[9,10]^. Decreases in HRV indicate less ability to maintain homeostasis, which may predispose the patient toward disease^[11,12]^.

Among the methods used for HRV analysis, quantitative and qualitative analysis of recurrence plots (RPs) are discussed here.

### Recurrence plot (RP)

According to Marwan et al.^[13]^, recurrence is a fundamental property of many dynamic systems and therefore various processes in nature. Eckmann et al.^[14]^ reported recurrence in a phase space that was termed a RP.

For the construction of RPs, one needs a time series generated by an experiment or numerical simulation on a computer. The RP is a highly effective and widely accepted tool for studying a time series^[15-21]^.

Its construction is very simple, being based on a square in which both the "x" axis and the "y" axis contain the elements of the time series, sequentially arranged from first to last. From this, according to the present value intervals between measurements (dimension) and distances or time intervals (radius), one can verify whether or not there are recurrence values^[21-23]^.

The use of different colors representing different radii complements the characteristic visual appearance of the graph. Rays with equal distances have the same color.

## METHODS

The study included 102 RR tachograms from the NUTECC database (Transdisciplinary Center for the Study of Chaos and Complexity), belonging to 45 patients (64.4% male) that underwent heart transplantation, regardless of the etiology of the disease. The tachograms were collected at rest, in the supine position, for about 20 minutes. In many cases, the collection was made a few minutes before performing an endomyocardial biopsy scheduled for follow-up.

The study was approved by the Research Ethics Committee of the Medical School of São José do Rio Preto, under number 251 021, on April 22, 2013.

Data collection was standardized^[11]^ as described below.

### The Recording of RR intervals

To evaluate the behavior of the time series, heart rate data were collected using a heart rate monitor called the Polar S810 that, according to Gamelin et al.^[24]^, shows good accuracy in the recording of low-intensity exercise when compared to the ambulatory electrocardiogram; it was also validated in Brazil^[26]^.

In this device, a belt with electrodes positioned at the patient's chest captures the electrical impulses of the heart and transmits them through an electromagnetic field to the monitor. The distress signal is sent via an interface with the Polar Precision Performance software, where units of time are fixed at 1 ms (millisecond) and samples of rate of recurrence (RR) intervals are collected at a frequency of 1000 Hz.

The RR interval series is analyzed and interference or artifacts are filtered in two stages:

(1) digitally, through the product's software; and
(2) manually, characterized by visual inspection of RR intervals and exclusion of abnormal intervals.

Only series with more than 95% regular beats are accepted.

For the analysis of the RR tachograms, a time series of 1000 RR intervals (data in milliseconds) was built, evaluating the following variables: Determinism (% DET), Recurrence (% REC), average diagonal length (Lmean), Shannon Entropy (ShEn), Sample Entropy (SampEn), and the RP qualitative visual aspect.

The RPs were built with the help of VRA 4.9 software, available for free on the Internet (http://visual-recurrence-analysis.software.informer.com) and developed by Eugene Kononov^[27]^. This software aims to assist researchers in qualitative and quantitative analysis of RPs. In this study, the parameters used in VRA 4.9 were: Embedding Dimension (M) = 10, Time Delay = 1, Radius = 70, and Line = 2; the color scheme was the "Volcano."

To perform the comparison of behavior patterns, mathematical models of random, chaotic, periodic, and linear time series were built. Those time series were analyzed by the VRA 4.9 software for visual and quantitative aspects.

The random series was built in Excel with the following formula: Random ( ) * 100, excluding decimal places and getting random values between 0 and 100.

The chaotic time series was built by the logistic equation: xt+1 = xt* (1-xt)*r, where xt = 0.2 and r = 3.7, which corresponds to known chaotic state. The values were also multiplied by 100, excluding the decimal place.

The periodic series was built by repeating the numbers 0-50, for a total of 521 numbers.

The linear series was constructed by means of a time series comprised of prime numbers from 2 to 3,800, with 521 numbers in total.

**Fig. 1:**
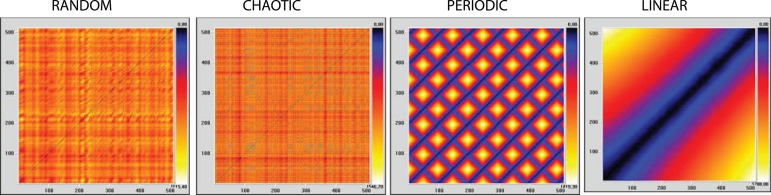
Recurrence plots (RP) of random, chaotic, periodic, and linear time series obtained by mathematical formulation.

Figure 1 shows the RPs carried out with the random, chaotic, periodic, and linear time series obtained by mathematical formulation.

VRA_m10_d1_L2_r70

Using those parameters, quantitative analysis of recurrence of random, chaotic, periodic and linear time series, obtained by mathematical formulation, as mentioned above, was performed (Table 1).

**Table 1:** Quantitative analysis of recurrence of time series: Random, Chaotic, Periodic, and Linear obtained by mathematical formulation.

	RR%	DET%	LAM%	TT	ENTR	Lmax
Random	6.2	87.1	20.2	2.327	2.879	25
Chaotic	20.1	96.4	0.8	3.297	4.118	82
Periodic	40.6	99.9	99.9	23.431	2.601	511
Linear	41.4	99.9	99.9	107.1	7.312	516

VRA_m10_d1_L2_r70 DET=determinism; ENTR=entropy; Lmax=diagonal maximum line; LAM=laminarity; RR=rate of recurrence; TT=Trapping Time

**Fig. 2:**
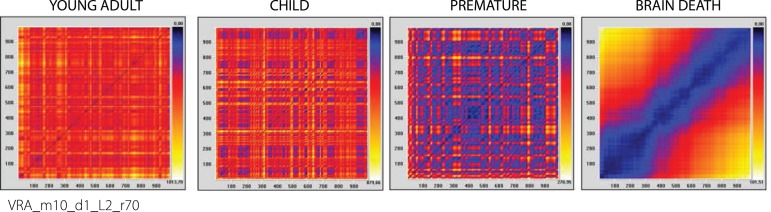
Recurrence plots (RP) of clinical time series: Young adult, Child, Premature newborn and Brain dead patient (data obtained from NUTECC).

RPs of clinical time series were also built to compare the behavior patterns of young adults (chaotic time series), children, premature newborns (time series tending to linearity), and brain dead patients (linear time series). Those plots are shown in Figure 2.

Table 2 shows the quantitative analysis of recurrence of clinical models performed with the same parameters of the mathematical models: RR, DET, diagonal maximum line (Lmax), entropy (ENTR), laminarity (LAM), and trapping time (TT).

**Table 2:** Quantitative analysis of recurrence of clinical time series: Young adult, Child, Premature newborn and Brain dead patient.

	RR%	DET%	Lam%	TT	ENTR	Lmax
Young adult	12.7	94.3	31.9	2.088	3.766	48
Child	18.6	95.6	90.1	5.193	3.884	76
Premature newborn	34.7	99.5	99.5	15.347	5.112	978
Brain dead patient	40.9	99.7	99.8	62.746	4.796	990

VRA_m10_d1_L2_r70 DET=determinism; ENTR=entropy; Lmax=diagonal maximum line; LAM=laminarity; RR=rate of recurrence; TT=Trapping Time

### Statistical Analysis

Descriptive analysis was performed (mean, standard deviation, median, and quartiles). Statistical calculations were done with the help of the StatsDirect statistical software, version 1.9.15 (30/11/2011). The hypothesis was that the extreme quartile values (first and fourth) of recurrence values should be associated , respectively, with greater and lesser follow-up times, since the variability of the heart rate should tend to increase with the passage of months. Continuous quantitative variables with Gaussian distribution were analyzed with unpaired Student's t-test or analysis of variance with Tukey post-test. Continuous quantitative variables, non-Gaussian and discrete quantitative variables were analyzed with the Mann-Whitney test. *P*-values ≤ 0.05 were considered statistically significant.

## RESULTS

The results are shown in Table 3.

**Table 3:** Descriptive analysis of the data (mean, standard deviation, and quartiles 1 and 4).

	Cutoff level (Q1)	Cutoff level (Q4)	Mean follow-up (months) ± standard deviation (Q1)	Mean follow-up (months) ± standard deviation (Q4)	*P*-value
%DET	99.05	99.82	43.31±35.42 (39.50)	40.13±36.399 (30)	0.7547
%REC	38.02	44.95	38.16±30.49 (37.59)	42.30±41.78 (22.53)	0.6905
Lmean (beats)	25.22	113.72	41.51±29.11 (37.96)	18.35±27.65 (4.605)	0.0059
ShEn	3.90	4.67	37.08±32.14 (3.54)	36.35±40.90 (13.55)	0.9452
SampEn	0.507	1.16	26.55±34 (27.96)	47.03±33.13 (13.55)	0.0361

%DET=determinism; %REC=recurrence; Lmean=average diagonal length; ShEn=Shannon entropy; SampEn=Sample entropy

The results of the comparison of the values of quartiles 1 and 4 of quantitative variables RPs showed statistical differences in Lmean and SampEn, with *P*=0.0059 and *P*=0.0361, respectively (Table 3).

**Fig. 3:**
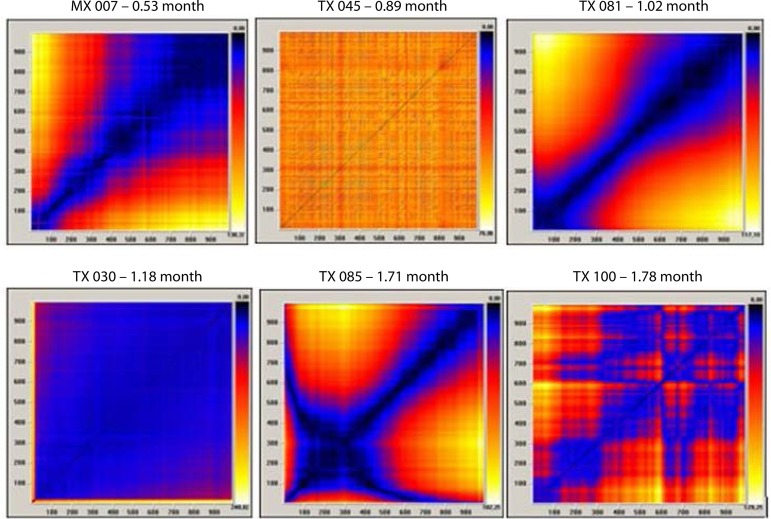
Recurrence plots (RP) of patients in the earlier postoperative period after heart transplantation (above each graph is the identification of the patient and the elapsed time of the transplant).

**Fig. 4:**
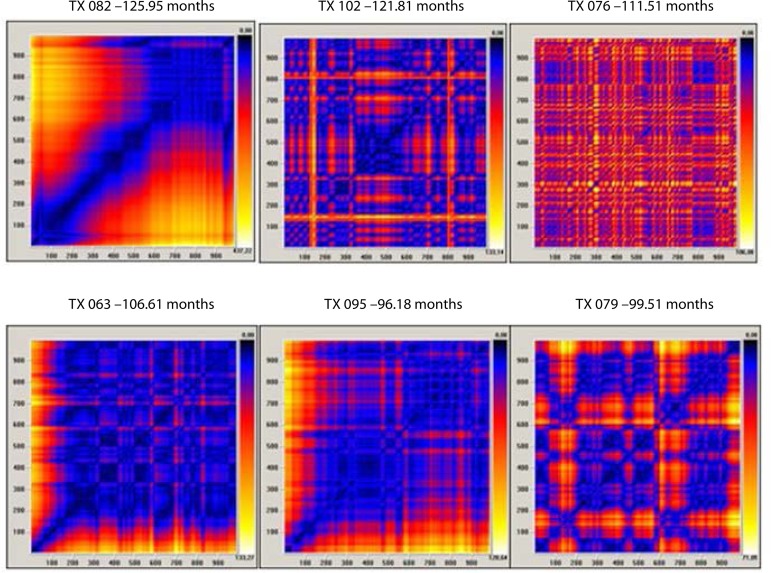
RP of patients in the later postoperative period after heart transplantation (above each graph is the identification of the patient and the elapsed time of the transplant).

Figures 3 and 4 show examples of the qualitative analysis of RP in some cardiac transplant patients.

When comparing RP built with the HRV time series of heart transplant patients with RPs of time series of mathematical (random, chaotic, periodic, and linear) and clinical models (young adult, child, premature newborn, and brain dead patient), certain similarity was found between:

- Heart transplant in more recent postoperative with linear mathematical model and brain dead patient clinical model.
- Heart transplant in later postoperative with periodic tending to chaotic mathematical model and premature newborn or child clinical models. There is an exception, TX 082, which resembles the linear mathematical model and brain death clinical model (the patient was coughing on the collection day. A few days after the collection, she was admitted to the intensive care unit with severe pneumonia.

## DISCUSSION

The results of the study showed that RPs may constitute good tools to assess HRV time series and, accordingly, analyze the autonomic nervous system and recovery in cardiac transplant patients.

Aparicio et al.^[27]^ reported that the quantitative analysis of recurrence is a technique of great interest because it allows us to make use of the advantages of RP and do so more objectively than deriving information purely from visual analysis.

Guo et al.^[28]^ analyzed recurrence variables in patients with coronary artery disease through morphological changes in pulse variability analysis. The aim of the study was to analyze if the pulse diagnosis, which is traditionally used in Chinese medicine, combined with a non-linear dynamic analysis could aid in the diagnosis of cardiovascular disease. They studied 37 patients with coronary artery disease and 37 healthy patients and recorded pulses, generating a time series. Patients remained at rest for five minutes and then pulse rates were collected by wrist measurement for 1 minute. The recurrence quantification analysis (RR, DET, Lmean, LMax, ShEn, LAM, and TT) showed significantly higher values for patients with coronary artery disease than in healthy individuals (*P*<0.05), translating into less variation and therefore higher autonomic physiological impairment.

Mohebbi and Ghassemian^[29]^ evaluated electrocardiogram segments of patients with atrial fibrillation (AF) in the 30 minutes preceding the arrhythmia and rated electrocardiogram segments spaced at least 45 minutes from any episode of paroxysmal AF. By comparing the RPs between the two groups, they found an increase in Lmax, Lmean, and ShEn in the segments preceding the occurrence of paroxysmal AF (*P*=0.0003 *P*=0.000006, and *P*=0.000008, respectively), showing that the episodes leading to paroxysmal AF are more stable (less chaotic) than the remote electrocardiogram episodes of AF. Visually, the qualitative assessment of RPs was similar to the patterns we observed, with a more homogeneous pattern in the segments of electrocardiogram prior to paroxysmal AF and more heterogeneous in the distant segments of paroxysmal AF^[29]^.

Melillo et al.^[30]^ studied HRV in 42 students under stress (during an oral test) and after a vacation period, with electrocardiogram recording for 5 minutes. The nonlinear analyses of HRV used were the Poincaré chart, approximate entropy (ApEn), correlation dimension (CD), Detrended fluctuations analysis (DFA), and RP. The HRV analysis measures showed different values in each situation (under stress and after vacation) and therefore can be used to differentiate low- and high-emotional stress situations^[30]^.

In the present study, Lmean values were higher in patients with low cardiac transplant time (*P*=0.0059), indicating that the average time that the two path segments remain similarly evolving into a system is significant, leading to greater average time system predictability and lower HRV and, consequently, lower performance of the autonomic nervous system in the hearts of these patients.

Tochigi et al.^[31]^ analyzed SampEn in 52 patients with symptomatic knee osteoarthritis and 57 asymptomatic controls, aged from 20-79 years. The hypothesis was that the variability of the leg movement in the affected knee could be smaller in older patients. The SampEn data in asymptomatic patients showed a significant negative correlation with increased age (r=-0.287, *P*=0.0306). Patients with osteoarthritis had significantly lower SampEn values (*P*=0.0002) than asymptomatic patients of the same age who walked with equivalent speed^[31]^.

Papaioannou et al.^[32]^ evaluated the respiratory complexity for weaning patients, using different nonlinear methods derived from the theory of complex systems, in a cohort of critically ill patients undergoing surgery. Thirty-two patients underwent the study, 22 of whom had typical weaning and 10 failed an evaluation for weaning. Tidal volume and average inspiratory flow were analyzed for 10 minutes during 2 phases: ventilation in pressure support of 15-20 cm H_2_O and evaluation for weaning pressure support of 5 cm H_2_O. SampEn was computed in the two respiratory phases in all patients and during the two stages of pressure support. Patients with weaning failure showed significantly decreased breathing pattern complexity, reflecting reduced SampEn of time series airflow, compared to patients with successful weaning (*P*<0.001). These results suggest that analyses of respiratory sign complexity may be important dynamic parameters to improve the prognosis of ventilator weaning in patients undergoing surgery^[32]^.

In the present study, we found that SampEn values were lower in transplant patients for less time and higher values in transplant patients were present for a longer period (*P*=0.0361) than in healthy controls. If there is less ENTR in information, there is less uncertainty and thus less variability.

The reduction in Lmean in the later segments also clearly indicates greater variability, so recurrences tend not to remain for long sequences.

Some non-linear measurements, such as DET, REC, and ShEn, did not show statistically significant differences between the earlier and the later groups of heart transplant patients. There are probably several peculiarities of the autonomic nervous system that we are not aware of in order to assess them. For example, we cannot define exactly whether the imbalance of the autonomic nervous system is associated with excess sympathetic action or a lack of parasympathetic action or the opposite. In addition, patients transplanted later start to present clinical complications related to the use of immunosuppressants, such as diabetes mellitus, hypertension, dyslipidemia, renal failure, osteoporosis, reactivation of Chagas disease, opportunistic infections, vascular disease graft, cancer, and coronary artery disease, which may be related to the worsening of autonomic nervous system homeostasis. Even if there is re-innervation of the heart in the medium and long terms, in these situations, diseases could evolve with greater RR interval linearity, leading to measures with low complexity.

The evolutionary qualitative analysis of the recurrence of the figure plots (Figures 1 and 2) tends toward a linear pattern, or toward training patterns over repetitive, less diffuse, and more homogeneous color characteristics when there is low variability and less time elapsed from transplantation. On the other hand, with more time elapsed from transplantation, there was a clear tendency to show a more diffuse pattern of heterogeneity of colors, suggesting a change in the linear reduction of meaning and, physiologically, partial return of autonomic control.

## CONCLUSION

The HRV analysis through RPs is a good tool to assess the re-innervation of the transplanted heart. There is evidence that heart re-innervation starts to happen approximately 18 months after transplantation, and improves gradually.

The RPs show, by means of the colors and patterns of the formed images, autonomic signs of recovery during follow-up in transplant patients. The RP may constitute a very promising method for monitoring heart transplant patients, which may indicate situations of decreased complexity and disease.

**Table:** 

Authors' roles & responsibilities	
ITT	Substantial contributions to the conception or design of the work; or the acquisition, analysis, or interpretation of data for the work; drafting the work or revising it critically for important intellectual content; agreement to be accountable for all aspects of the work in ensuring that questions related to the accuracy or integrity of any part of the work are appropriately investigated and resolved; final approval of the version to be published
RAH	Drafting the work or revising it critically for important intellectual content; agreement to be accountable for all aspects of the work in ensuring that questions related to the accuracy or integrity of any part of the work are appropriately investigated and resolved; final approval of the version to be published
MAS	Drafting the work or revising it critically for important intellectual content; agreement to be accountable for all aspects of the work in ensuring that questions related to the accuracy or integrity of any part of the work are appropriately investigated and resolved; final approval of the version to be published
FCP	Drafting the work or revising it critically for important intellectual content; agreement to be accountable for all aspects of the work in ensuring that questions related to the accuracy or integrity of any part of the work are appropriately investigated and resolved; final approval of the version to be published
JHN	Drafting the work or revising it critically for important intellectual content; agreement to be accountable for all aspects of the work in ensuring that questions related to the accuracy; final approval of the version to be published
DLG	Drafting the work or revising it critically for important intellectual content; agreement to be accountable for all aspects of the work in ensuring that questions related to the accuracy or integrity of any part of the work are appropriately investigated and resolved; final approval of the version to be published
VFN	Drafting the work or revising it critically for important intellectual content; agreement to be accountable for all aspects of the work in ensuring that questions related to the accuracy or integrity of any part of the work are appropriately investigated and resolved; final approval of the version to be published
TQF	Drafting the work or revising it critically for important intellectual content; agreement to be accountable for all aspects of the work in ensuring that questions related to the accuracy or integrity of any part of the work are appropriately investigated and resolved; final approval of the version to be published
GCC	Drafting the work or revising it critically for important intellectual content; agreement to be accountable for all aspects of the work in ensuring that questions related to the accuracy or integrity of any part of the work are appropriately investigated and resolved; final approval of the version to be published
MFG	Substantial contributions to the conception or design of the work; or the acquisition, analysis, or interpretation of data for the work; drafting the work or revising it critically for important intellectual content; agreement to be accountable for all aspects of the work in ensuring that questions related to the accuracy or integrity of any part of the work are appropriately investigated and resolved; final approval of the version to be published
